# Evaluation of TNF-α cytokine production in patients with tuberculosis compared to healthy people

**DOI:** 10.3205/dgkh000315

**Published:** 2018-11-16

**Authors:** Arezou Mirzaei, Hassan Mahmoudi

**Affiliations:** 1 Islamic Azad University, Hamadan Branch, Hamadan, Iran; 2 Department of Microbiology, Hamadan University of Medical Sciences, Hamadan, Iran

**Keywords:** mycobacterium tuberculosis, tumor necrosis factor

## Abstract

**Background:** *Mycobacterium tuberculosis* (TB) is one of the most important causes of human mortality. Approximately one-third of the world’s population is infected with TB and 5–10% of them develop the active form of the disease. Cytokines play a major role in the host defense process against Mycobacterium infections. Among these cytokines, tumor necrosis factor alpha (TNF-α) has a prominent role in the defense of and pathological responses to tuberculosis.

**Materials and methods:** A case-control study was carried out from May 2016 to June 2017. 45 patients with diagnosis of tuberculosis (smear and positive culture) were included as case group and 45 healthy subjects as control group. The serum levels of TNF-α were determined with the enzyme-linked immunosorbent assay (ELISA) method.

**Results:** The concentration of TNF-α in patients with TB was significantly higher than in the control group (P<0.05). However, the difference was only significant in the age groups 20–30 and 50–60 years; in the age groups 30–40, 40–50 and 50–70 years, the difference was not significant, although certain trends were apparent.

**Discussion and conclusion:** Since the level of serum TNF-α is higher in patients with pulmonary tuberculosis than in individuals without it, the measurement of TNF-α levels can be useful as a probable marker for the diagnosis of tuberculosis.

## Zusammenfassung

**Hintergrund:** *Mycobacterium tuberculosis* (TB) ist eine der wichtigsten Ursachen der menschlichen Sterblichkeit. Ungefähr 1/3 der Weltbevölkerung ist mit TB infiziert; davon erkranken 5–10%. Zytokine spielen eine wichtige Rolle im Abwehrprozess gegen Mycobacterium-Infektionen. Unter den Zytokinen besitzt der Tumornekrosefaktor (TNF-α) eine herausragende Rolle bei der Abwehr und bei pathologischen Reaktionen auf Tuberkulose.

**Material und Methode:** Die Fall-Kontroll-Studie wurde vom Mai 2016 bis Juni 2017 durchgeführt. Es wurden 45 Patienten mit der Diagnose Tuberkulose (Abstrich und positive Kultur) als Fallgruppe und 45 gesunde Probanden als Kontrollgruppe untersucht. Die Serumspiegel von TNF-α wurden mittels ELISA bestimmt.

**Ergebnisse:** In der Fallgruppe war die Konzentration von TNF-α signifikant höher als in der Kontrollgruppe (P<0,05). Der Unterschied war jedoch nur in den Altersgruppen 20–30 und 50–60 Jahre signifikant; in den Altersgruppen 30–40, 40–50 und 50–70 Jahre war die Differenz nur tendenziell.

**Diskussion und Schlussfolgerung:** Da bei Patienten mit Lungentuberkulose der TNF-α Serumspiegels erhöht ist, kann seine Bestimmung als möglicher zusätzlicher Marker für die Diagnose der Tuberkulose nützlich sein.

## Introduction

*Mycobacterium tuberculosis* (TB) is one of the most important causes of mortality in humans. According to the World Health Organization report in 2017, tuberculosis is responsible for 1.7 million deaths every year [1]. About one-third of the world population is infected, but only about 10% of these develop active tuberculosis [2]. The causative agent is the intracellular bacterial pathogen itself; its antigens have the ability to stimulate the production of cytokines via the mononuclear phagocyte system. Studies show that susceptibility to the disease varies among individuals, e.g., not everyone who is exposed to this bacterium becomes infected with TB [3]. The course of the disease also differs from person to person. These differences can be due to host factors and the genetic sensitivity of various individuals [3]. In this disease, cellular immunity and cytokines are intermediaries in the immune system and inflammatory responses [4], [5]. It seems that the acquired immune response in the pathway of T-helper lymphocytes can limit and stop bacterial growth. These cells secrete cytokines, such as interferon gamma and TNF-α, and stimulate macrophages to further produce more active oxygen and free radicals. As a result, cells are better able to kill microbes, and more microbial antigens are delivered to T cells [6], [7]. T-helper 2-type cytokines such as IL-4, IL-5, IL-10, IL-13 influence the course of the disease [8]. Tumor Necrosis Factor-α is a cytokine produced by many types of cells, such as macrophages and monocytes. TNF-α exists in two forms; a type II transmembrane protein, and a mature soluble protein. However, this cytokine is initially produced as a membrane protein of 212 amino acids [9]. TNF-α binding to cell surface receptors TNFRp55 (TNF-R1, CD120a or p55/60) and TNF Rp75 (aR2, CD120b and p75/80) triggers an intracellular signaling cascade. The Rp55 receptor is expressed by most tissues, and TNF-α terraform solution is mainly attached to Rp55 receptor, although activated by both types of TNF-α. The membrane associated form mostly binds to TNF Rp75, which is specific to the immune system cells [10]. Awareness of the specific functions and the activation of TNF-α receptors considerably help to understand the inhibitory effects of TNF-α on some diseases [11]. The purpose of this study was to determine the importance of TNF-α in protecting against tuberculosis and investigate the rate of production of this cytokine in patients with tuberculosis as compared to the control group.

## Materials and methods

### Study design

A case-control study was conducted from June 2016 to July 2017. The case group consisted of 45 patients with a definite tuberculosis diagnosis (with smear and positive culture), and 45 healthy subjects were selected as the control group.

### Measurement of TNF-α

TNF-α was measured using the enzyme-linked immunosorbent assay (ELISA) (Human TNF-α Platinum ELISA Kit [96Test], eBayscience Company ([BMS223/4CE], [BMS223/4TENCE]).

### Statistical analysis

ANOVA, Tukey’s test, and Bonferroni correction were employed, as was the Shapiro-Wilk test (P≤0.05) due to the low sample size.

## Results

**Figure 1:**
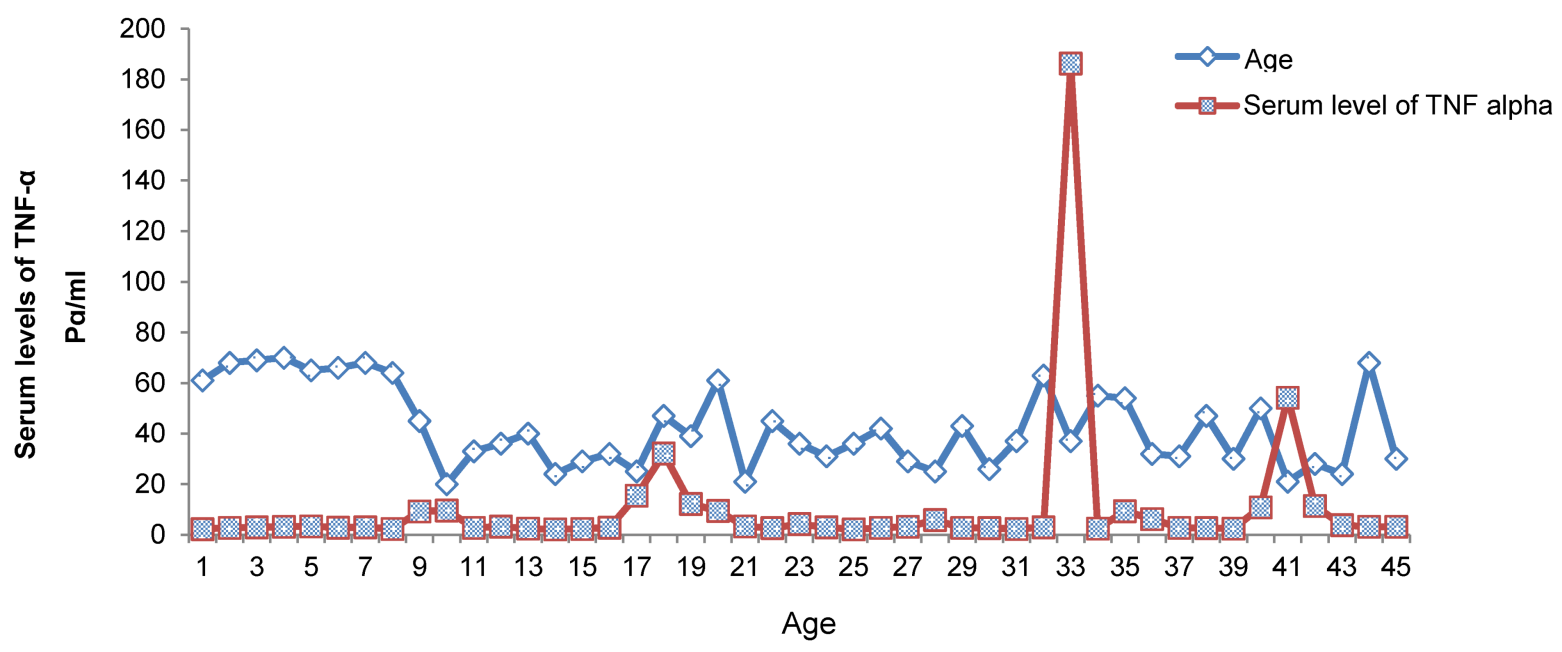
Serum levels of TNF-α in the tuberculosis patient group (case group)

**Figure 2:**
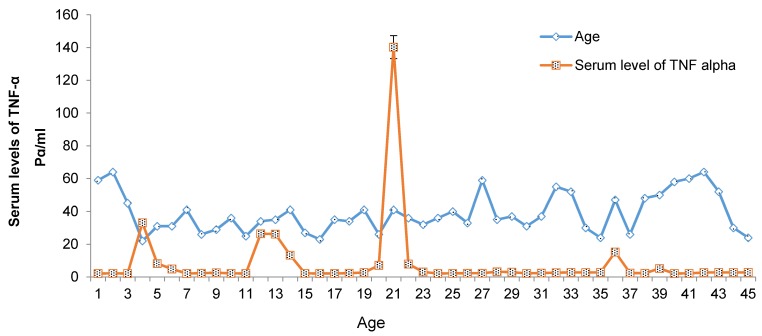
Serum levels of TNF-α in the control group

The age of patients ranged from 20 to 60 years, with a mean age of 41±1.5 years. The study population included 52 (68.8%) men and 38 (42.2%) women. The mean serum level of TNF-α in the patient group were 10.6 pg/ml with the lowest and highest rates being 1.2 and 186.2 pg/ml, respectively. The mean serum level of TNF-α in the control group was 8.26 pg/ml, with the lowest and highest rates being 1.2 and 240 pg/ml, respectively (Figure 1 (Fig. 1), Figure 2 (Fig. 2)). 

**Figure 3:**
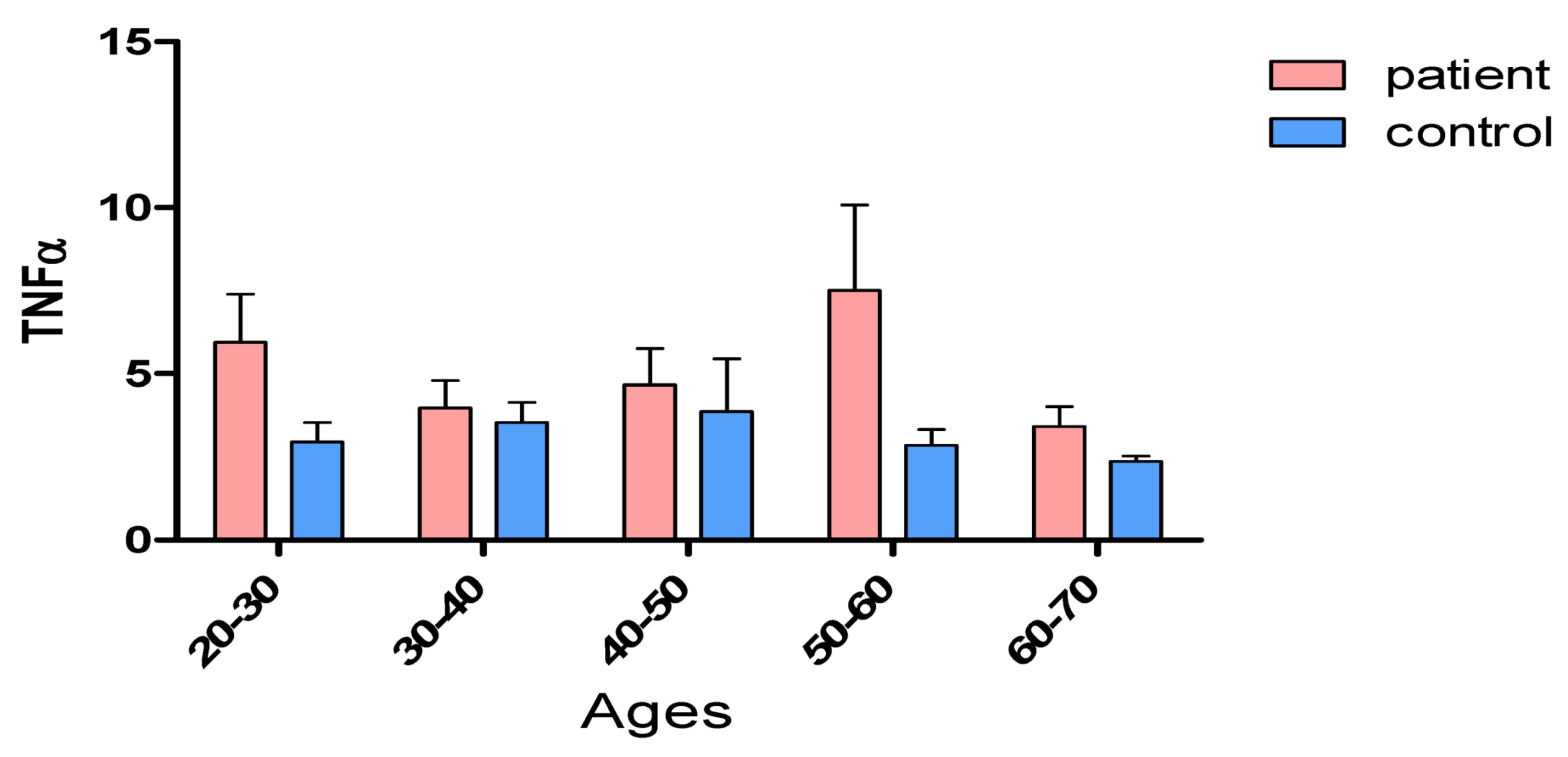
Correlation between serum levels of TNF-α and different age groups

Data were evaluated in Prism 6 GraphPad software, using one-way ANOVA and Tukey’s tests. A comparison of serum levels of TNF-α with different age groups is shown in Figure 3 (Fig. 3). The results show that between the control group and the patient group, there was a significant difference (P<0.05) in the age groups 20–30 and 50–60 years of age. There were no significant differences in the other age groups (Table 1 (Tab. 1)). 

## Discussion

Pulmonary tuberculosis is one of the most important infectious diseases in humans. Despite the use of anti-tuberculosis drugs, this disease is one of the major causes of mortality, especially in developing countries. In recent years, researchers attempted to find a link between serum cytokines TNF-α, IFN-γ, IL-12, IL-10, and *Mycobacterium tuberculosis*. The results of this study showed that TNF-α production in patients with *Mycobacterium tuberculosis* infection was greater than serum levels of TNF-α in the control group. Cytokines play a crucial role in the host defense process against *Mycobacterium* infections [12]. Among these cytokines, TNF-α has a prominent role in defense and pathological responses to tuberculosis [13], [14]. In a study by Anoosheh et al. [12], cytokine gene polymorphisms in PTB and healthy controls were evaluated. The results demonstrated the importance of cytokine TNF-α. The findings of other authors also showed a significant correlation between patient and control groups (P<0.05) [11]. In this study, serum levels of TNF-α were significantly higher in the patient group than in the control group. Our findings suggest that serum levels of TNF-α increase in patients with pulmonary tuberculosis. Fatima et al. [15] studied the serum level of TNF-α in patients with tuberculosis. The results of their study, which were similar to those of the present study, showed a significant increase in serum levels of TNF-α in the patient group compared to the control group (P<0.001). Other studies have shown the importance of TNF-α in patients with tuberculosis [16], [17], [18]. The results of these studies showed that the serum levels of TNF-α decreased significantly with treatment. Nakya et al. [19] studied serum levels of TNF-α in patients with active pulmonary tuberculosis compared to a control group. The results of that study showed that the serum level of TNF-α increased significantly in the patient group compared with the control group, which agrees with our results (P<0.001). In the study by Shameem et al. [20], serum levels of TNF-α were investigated in TB patients. The results did not demonstrate a significant relationship between the control group and the patients, which contradicts the results of this study (P<0.05). In another study [21], the serum levels of TNF-α in *Mycobacterium tuberculosis* and non-tuberculosis diseases were measured. The results of that study showed that the serum levels of TNF-α in patient with *Mycobacterium tuberculosis* did not differ significantly compared to healthy controls. This contradicts the results of the current study. In another study, the serum level of TNF-α was measured in peripheral blood monocytes of Mycobacterium and control patients [22]. The difference between results from various studies is correlated with the geographic location of the samples and the type of Mycobacteria prevalent in the studied area.

## Conclusion

The findings of this study show that cytokines can be used as a possible marker for the diagnosis of tuberculosis. Measuring the level of cytokines can be used to supplement the diagnostic tests for Mycobacterium. More studies are needed to confirm these findings.

## Notes

### Competing interests

The authors declare that they have no competing interests.

## Figures and Tables

**Table 1:**
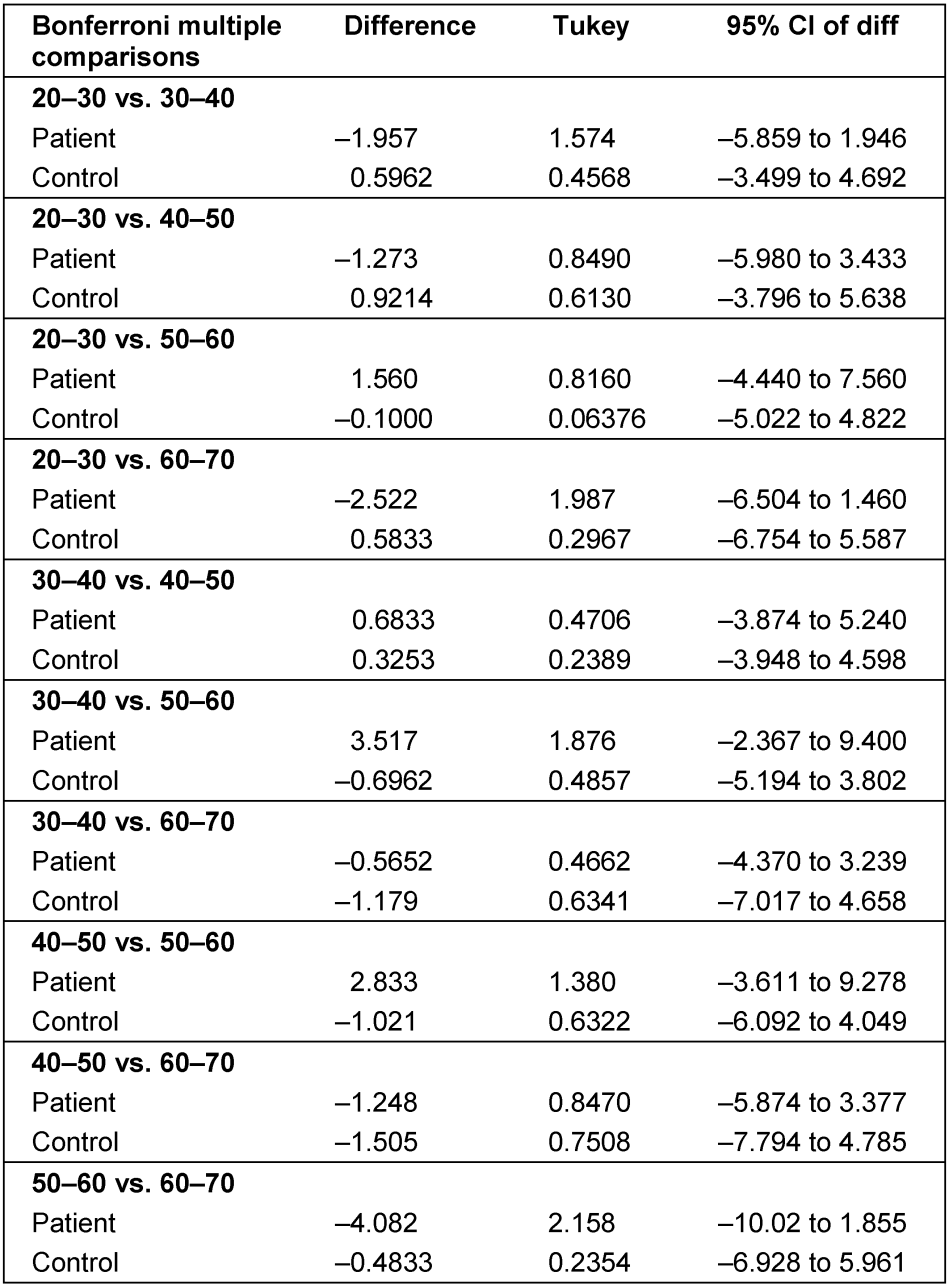
Analysis of TNF-α serum levels by age groups

## References

[1] Mulu W, Abera B, Yimer M, Hailu T, Ayele H, Abate D (2017). Rifampicin-resistance pattern of Mycobacterium tuberculosis and associated factors among presumptive tuberculosis patients referred to Debre Markos Referral Hospital, Ethiopia: a cross-sectional study. BMC Res Notes.

[2] Asgharzadeh M, Ghorghanlu S, Rashedi J, Mahdavi Poor B, Khaki-Khatibi F, Moaddab SR, Samadi-Kafil H, Pourostadi M (2016). Association of Promoter Polymorphisms of Interleukin-10 and Interferon-Gamma Genes with Tuberculosis in Azeri Population of Iran. Iran J Allergy Asthma Immunol.

[3] Moran A, Ma X, Reich RA, Graviss EA (2007). No association between the +874T/A single nucleotide polymorphism in the IFN-gamma gene and susceptibility to TB. Int J Tuberc Lung Dis.

[4] Huang W, Na L, Fidel PL, Schwarzenberger P (2004). Requirement of interleukin-17A for systemic anti-Candida albicans host defense in mice. J Infect Dis.

[5] Karaoglan I, Pehlivan S, Namiduru M, Pehlivan M, Kilinçarslan C, Balkan Y, Baydar I (2009). TNF-alpha, TGF-beta, IL-10, IL-6 and IFN-gamma gene polymorphisms as risk factors for brucellosis. New Microbiol.

[6] Biranvand E, Abedian Kenary S, Ghaheri A, Rezaee MS, Hasannia H, Nasrolahi M, Parsaee MR, Ahanjan M, Biranvand B, Ahmadi Basiri E (2011). Medical Laboratory Journal (goums).

[7] Galanakis E, Makis A, Bourantas KL, Papadopoulou ZL (2002). Interleukin-3 and interleukin-4 in childhood brucellosis. Infection.

[8] Fernandes DM, Baldwin CL (1995). Interleukin-10 downregulates protective immunity to Brucella abortus. Infect Immun.

[9] Aung H, Toossi Z, Wisnieski JJ, Wallis RS, Culp LA, Phillips NB, Phillips M, Averill LE, Daniel TM, Ellner JJ (1996). Induction of monocyte expression of tumor necrosis factor alpha by the 30-kD alpha antigen of Mycobacterium tuberculosis and synergism with fibronectin. J Clin Invest.

[10] Ehlers S (2003). Role of tumour necrosis factor (TNF) in host defence against tuberculosis: implications for immunotherapies targeting TNF. Ann Rheum Dis.

[11] Mootoo A, Stylianou E, Arias MA, Reljic R (2009). TNF-alpha in tuberculosis: a cytokine with a split personality. Inflamm Allergy Drug Targets.

[12] Anoosheh S, Farnia P, Kargar M, Kazem Poor M, Seif S, Noroozi J, Masjedi MR, Velayati A (2009). Majallahi Ilmi Pizhuhishii Danishgahi Ulumi Pizishki Khadamati Bihdashtii Darmanii Zanjan.

[13] Oh JH, Yang CS, Noh YK, Kweon YM, Jung SS, Son JW, Kong SJ, Yoon JU, Lee JS, Kim HJ, Park JK, Jo EK, Song CH (2007). Polymorphisms of interleukin-10 and tumour necrosis factor-alpha genes are associated with newly diagnosed and recurrent pulmonary tuberculosis. Respirology.

[14] Giacomini E, Iona E, Ferroni L, Miettinen M, Fattorini L, Orefici G, Julkunen I, Coccia EM (2001). Infection of human macrophages and dendritic cells with Mycobacterium tuberculosis induces a differential cytokine gene expression that modulates T cell response. J Immunol.

[15] Nazish Fatima, Mohammad Shameem, Nabeela, Khan HM (2016). Cytokines as Biomarkers in the Diagnosis of MDR TB Cases. EC Pulmonology and Respiratory Medicine.

[16] Tang S, Xiao H, Fan Y, Wu F, Zhang Z, Li H, Yang Y (2002). Zhonghua Jie He He Hu Xi Za Zhi.

[17] Portales-Pérez DP, Baranda L, Layseca E, Fierro NA, de la Fuente H, Rosenstein Y, González-Amaro R (2002). Comparative and prospective study of different immune parameters in healthy subjects at risk for tuberculosis and in tuberculosis patients. Clin Diagn Lab Immunol.

[18] Kawaguchi H, Ina Y, Ito S, Sato S, Sugiura Y, Tomita H, Ogisu N, Takada K, Yamamoto M, Morishita M, Yoshikawa K (1996). Kekkaku.

[19] Nakaya M, Yoneda T, Yoshikawa M, Tsukaguchi K, Tokuyama T, Fu A, Okamoto Y, Fukuoka K, Yamamoto C, Fukuoka A (1995). Kekkaku.

[20] Shameem M, Fatima N, Khan HM (2015). Association of TNF-α serum levels with response to antitubercular treatment in MDR tuberculosis patients. Annals of Tropical Medicine and Public Health.

[21] Taghavi K, Farnia P, Anooshe S, Bayat M, Kazempoor M, Masjedi M, Velayati A (2010). Journal of Shahid Sadoughi University of Medical Sciences.

[22] Shahemabadi AS, Hosseini AZ, Shaghsempour S, Masjedi MR, Rayani M, Pouramiri M (2007). Evaluation of T cell immune responses in multi-drug-resistant tuberculosis (MDR-TB) patients to Mycobacterium tuberculosis total lipid antigens. Clin Exp Immunol.

